# Towards practical time-of-flight secondary ion mass spectrometry lignocellulolytic enzyme assays

**DOI:** 10.1186/1754-6834-6-132

**Published:** 2013-09-14

**Authors:** Robyn E Goacher, Alex Yi-Lin Tsai, Emma R Master

**Affiliations:** 1 Department of Biochemistry, Chemistry and Physics, Niagara University, Niagara University, NY, USA; 2 Department of Cell and Systems Biology, University of Toronto, Toronto, Ontario, Canada; 3 Department of Chemical Engineering and Applied Chemistry, University of Toronto, Toronto, Ontario, Canada

**Keywords:** ToF-SIMS, Lignocellulose, Enzymes, Extractives, Cellulase

## Abstract

**Background:**

Time-of-Flight Secondary Ion Mass Spectrometry (ToF-SIMS) is a surface sensitive mass spectrometry technique with potential strengths as a method for detecting enzymatic activity on solid materials. In particular, ToF-SIMS has been applied to detect the enzymatic degradation of woody lignocellulose. Proof-of-principle experiments previously demonstrated the detection of both lignin-degrading and cellulose-degrading enzymes on solvent-extracted hardwood and softwood. However, these preliminary experiments suffered from low sample throughput and were restricted to samples which had been solvent-extracted in order to minimize the potential for mass interferences between low molecular weight extractive compounds and polymeric lignocellulose components.

**Results:**

The present work introduces a new, higher-throughput method for processing powdered wood samples for ToF-SIMS, meanwhile exploring likely sources of sample contamination. Multivariate analysis (MVA) including Principal Component Analysis (PCA) and Multivariate Curve Resolution (MCR) was regularly used to check for sample contamination as well as to detect extractives and enzyme activity. New data also demonstrates successful ToF-SIMS analysis of unextracted samples, placing an emphasis on identifying the low-mass secondary ion peaks related to extractives, revealing how extractives change previously established peak ratios used to describe enzyme activity, and elucidating peak intensity patterns for better detection of cellulase activity in the presence of extractives. The sensitivity of ToF-SIMS to a range of cellulase doses is also shown, along with preliminary experiments augmenting the cellulase cocktail with other proteins.

**Conclusions:**

These new procedures increase the throughput of sample preparation for ToF-SIMS analysis of lignocellulose and expand the applications of the method to include unextracted lignocellulose. These are important steps towards the practical use of ToF-SIMS as a tool to screen for changes in plant composition, whether the transformation of the lignocellulose is achieved through enzyme application, plant mutagenesis, or other treatments.

## Background

The utilization of plant matter (lignocellulose) to create 2nd-generation biofuels and value-enhanced bioproducts is one of several approaches that will be required to supplant our existing reliance on fossil fuels and petroleum products. Due to their specificity and gentle reaction conditions, enzymatic processes are important to these efforts. Recent advances in enzyme discovery have been enabled by genome sequencing. However, robust screens to assess enzyme action on relevant biomass substrates remain limiting.

Time-of-Flight Secondary Ion Mass Spectrometry (ToF-SIMS) is a surface mass spectrometry technique with potential advantages for the study of lignocellulosic materials. These advantages include direct analysis of complex solid substrates, the potential to monitor both lignocellulose degradation and modification, and the potential for fast enzyme assays due to the technique’s surface sensitivity [1]. ToF-SIMS has been applied for some years to the analysis of lignin and extractives on pulp and paper surfaces [2-7], making particular use of the imaging ability of ToF-SIMS and of the analysis of molecular fragments. Applications of ToF-SIMS to lignocellulose include mapping of lignin, polysaccharide, extractive and inorganic components [8-14], evaluation of pretreatment methods for hydrolysis of poplar [15] and birch [16], and studies of the enzymatic and fungal degradation of wood [1,17].

Previously, we have identified 40 secondary ion peaks characteristic of lignin and polysaccharides in extracted pine [18] and have established good reproducibility of lignin and polysaccharide peak ratios across powdered wood samples [1]. This peak list was further refined to exclude mass interferences from applied proteins, leading to ToF-SIMS assays to measure cellulase and laccase action on solvent-extracted spruce and aspen [1].

The assessment of enzyme activity by ToF-SIMS is approached through relative quantification of peaks characteristic of lignin and polysaccharides. It was observed, for example, that cellulase treatment resulted in the degradation and dissolution of polysaccharides from the wood sample, producing a lignin-rich surface residue [1]. In a separate study, a combination of ToF-SIMS and infrared spectroscopy was also used to elucidate the mechanism of fungal degradation of two coniferous wood samples [17]. Similar to previous assessments of enzyme activity using ToF-SIMS, in this study, wood samples were solvent-extracted after fungal degradation and prior to ToF-SIMS analysis [17].

While ToF-SIMS is a promising technique for characterizing lignocellulose-active enzymes, several limitations in currently published methods must be overcome to achieve higher sample throughput on broader sample types. These limitations reflect efforts to simplify sample spectra by 1) carefully handling each sample to avoid surface contamination, and 2) solvent-extracting the wood samples before enzyme treatment. For instance, in previous analyses, wood samples were incubated in glass vials and processed manually [1,17,18]. Moreover, given the potential of certain extractive compounds to form fragment ions similar to lignin, wood samples were solvent-extracted to reduce the likelihood of mass interferences that could complicate the interpretation of ToF-SIMS data.

Automated sample handling would speed up sample washing and the transfer of solid samples to supports for processing by ToF-SIMS. By avoiding the solvent extraction of wood samples, enzymes could be screened on more commercially relevant feedstocks and enzymes resistant to wood extractives could also be discovered. Accordingly, the current study 1) introduces a new higher-throughput method for handling lignocellulose powder for transfer to ToF-SIMS; 2) identifies low-mass secondary ion peaks related to plant extractives, and 3) tests whether enzyme activity can be measured in the presence of extractives.

Previous ToF-SIMS enzyme assays applied a commercial cellulase cocktail and laccase sample [1], but were not yet extended to novel enzyme screening. Therefore, in addition to expediting sample preparation, ToF-SIMS based enzyme screens were further developed by demonstrating the potential to discover carbohydrate-active-enzymes (CAZymes) through cocktail augmentation. Specifically, a dose–response curve for a commercial cellulase cocktail is demonstrated, and preliminary cocktail augmentation experiments are shown as the next step to screening novel proteins. Finally, improvements in sample handling and treatment are combined by assessing the reproducibility of cellulase dose response for extracted and unextracted wood substrates.

## Results and discussion

### Increased sample throughput using 96-well filter plates and tape support

To increase sample processing throughput, enzyme incubation and sample washing were switched from glass scintillation vials to 96-well filter plates. Overall, the use of the 96-well filter plates for sample incubation and washing, combined with transferring washed biomass samples to tape for support in the ToF-SIMS, resulted in significant time and material savings compared to previous methods [1] (Table 1). More specifically, miniaturization of the enzyme assays to 96-well format reduced enzyme and wood sample quantities to 25% of earlier requirements [1]. Washing samples on the 96-well plates using vacuum filtration also reduced washing times by 75%, and transfer of the wood powder to the tape support took 10 minutes rather than the 4 h it would have taken to prepare 96 die-pressed wood pellets [1]. The simple process of transferring air-dried wood powder from the wells within a filter plate to a tape support is illustrated in Additional file 1: Figure S1. Notably, given the 96-well format of sample preparation, this improved procedure could be automated using high-throughput liquid handling robotics.

**Table 1:** Time and material savings using 96-well filter plates

**Original assays in 3 mL glass vials**[1]	**Assays using 96-well filter plates**
15–20 mg of wood per vial	4–5 mg of wood per well
1–2 mL of solution per vial	250 μL of solution per well
Manual sample washing was tedious, requiring settling and pipetting times exceeding 2 h for 96 samples	Whole plate is washed in parallel using a vacuum manifold (20–30 min, robotic or manual for 96 wells)
Wood fibers were individually pressed into 13-mm pellets and mounted for ToF-SIMS, a 4 h process for 96 samples	Wood fibers are transferred to adhesive tape for ToF-SIMS analysis, a 10 minute process for all 96 wells

### Confirming absence of sample contamination by 96-well filter plates

**Figure 1:**
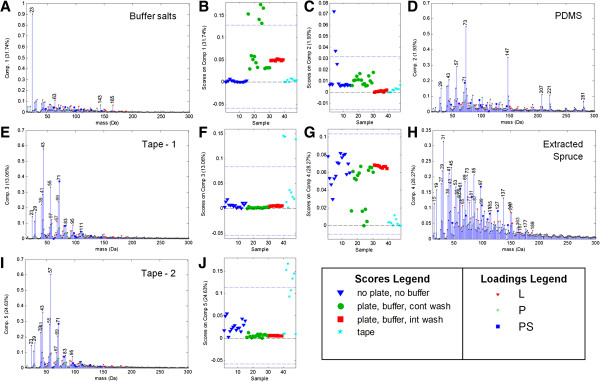
**MCR model describing sample contamination, washing and tape support for samples treated in 96-well filter plates.** Samples in the model included Scotch® tape alone, extracted spruce that had not been exposed to the filter plate or to buffer (“no plate, no buffer”), and extracted spruce incubated in buffer on the 96-well filter plate and then washed on-plate with either continuous or intermittent vacuum (“plate, buffer, cont wash” or “plate, buffer, int wash”). Scores plots **(B**,**C**,**F**,**G**,**J)** show how well the positive ion ToF-SIMS spectra match each pure component spectrum describing buffer salts **(A)**, PDMS contamination **(D)**, tape **(E**,**I)** and spruce **(H)**.

Since the spectral sensitivity of ToF-SIMS ranges from ppm to ppb, the potential for surface contamination by plasticizers (e.g. phthalates or poly(dimethyl siloxane), PDMS) potentially used in the 96-well plates was a concern. To address the possibility that 96-well filter plates may impart contaminants to wood powder preparations, ToF-SIMS spectra of Soxhlet-extracted spruce powder before and after exposure to the filter plates were visualized using a multivariate curve resolution (MCR) model (Figure 1). This model also revealed residual buffer components and adhesive tape, which are discussed separately below. MCR is a useful statistical method for extracting pure component spectra from complicated ToF-SIMS data sets, as more fully described in Ref [19]. Each individual spectrum is described as a combination of these pure components by the spectrum’s scores on each component.

Over the course of many experiments using the filter plates, no evidence of phthalate contamination was observed. While poly(dimethyl siloxane), PDMS, was observed on some samples, similar incidence of PDMS contamination was observed when handling wood samples with and without exposure to the filter plate (Figure 1C,D). The PDMS is therefore likely to come from sources aside from the filter plates, suggesting that the filter plates would be suitable for ToF-SIMS sample preparation.

The clear PDMS spectrum in the MCR model illustrates the value of routinely analyzing full spectral data by multivariate analysis to screen for contaminants. Such routine data screening has occasionally revealed other localized contaminants in other experiments (data not shown), emphasizing the good practice of distributing biological replicates of enzyme assays to distant areas of the filter plate, and pre-extracting the filter plates with reaction buffer and/or appropriate solvents.

### On-plate sample washing

Incubation in buffer is often necessary for optimal enzyme activity. However, salts cause several negative effects in ToF-SIMS: they can mask other signals from the samples due to mass interference and they may also cause matrix effects, changing ion yields of the lignocellulose-related ions. Two on-plate sample washing methods were tested to remove buffer salts. Samples were incubated with universal buffer and then washed with distilled water, applying suction to the filter plate either continuously or intermittently.

A ToF-SIMS peak pattern related to sodium (23 Da) was observed in the MCR model of the samples exposed to buffer (Figure 1A,B). The samples continuously washed with water exhibited the highest salt contents (highest scores on comp. 1), followed by samples washed intermittently. The peak at 23 Da was comparatively low or non-detectable in samples that were not exposed to buffer. It was concluded that despite the large volume of wash water used, the application of continuous vacuum during washing did not permit sufficient circulation of the particles in the rinse water. Therefore, the washing procedure was altered to use intermittent vacuum and re-suspension of the wood pellet was encouraged by either forceful addition of wash water with the pipette and/or multi-aspiration. To provide enough fresh wash water, several washing aliquots were used, with ~1 minute rest time between each suction cycle. This resulted in better removal of buffer salts, as demonstrated by the lower scores on comp. 1 for the “*plate, buffer, int. wash*” samples (Figure 1A,B).

For the buffer-exposed samples, the abundance of the Na^+^ ion as a percentage of the total spectrum intensity (*Na*^*+*^*/total ions*) ranged from 5-36%. Above ~20% Na^+^, many inorganic salt clusters were observed to contribute significantly to the spectrum. The most abundant of these were at 62.98 Da (Na_2_OH^+^), 124.94 Da (Na_2_PO_3_^+^), 142.95 Da (Na_2_H_2_PO_4_^+^) and 164.93Da (Na_3_HPO_4_^+^), however, new inorganic peaks on the mass-deficit side of the nominal mass were observed throughout the spectra.

### Adhesive tape as a sample support

Several ToF-SIMS spectra of the pure adhesive tape used to support the spruce powder were also collected and included in the MCR model (Figure 1). Interestingly, there were two different pure component spectra found to describe the tape (Figure 1E,F and Figure 1I,J). Most of the samples did not score highly on these components, but the “*no plate, no buffer*” extracted spruce clearly scored higher on these tape components, indicating incomplete coverage of the spruce particles on the tape, which was also visible optically. Some peaks previously identified as characteristic of lignocellulose [1] did overlap with peaks in the tape spectrum – most predominately at 71 Da but also significantly at 67, 79 and 81 Da.

It is important to recognize this tape pattern using multivariate analysis prior to interpreting peak ratios since the increased tape show-through resulted in a slight increase of the polysaccharide peak fraction [1] from 0.50 to 0.56 and a large decrease of the lignin modification metric [1] from 0.92 to 0.68 (Additional file 1: Table S1).

### Peaks related to extractives

**Figure 2:**
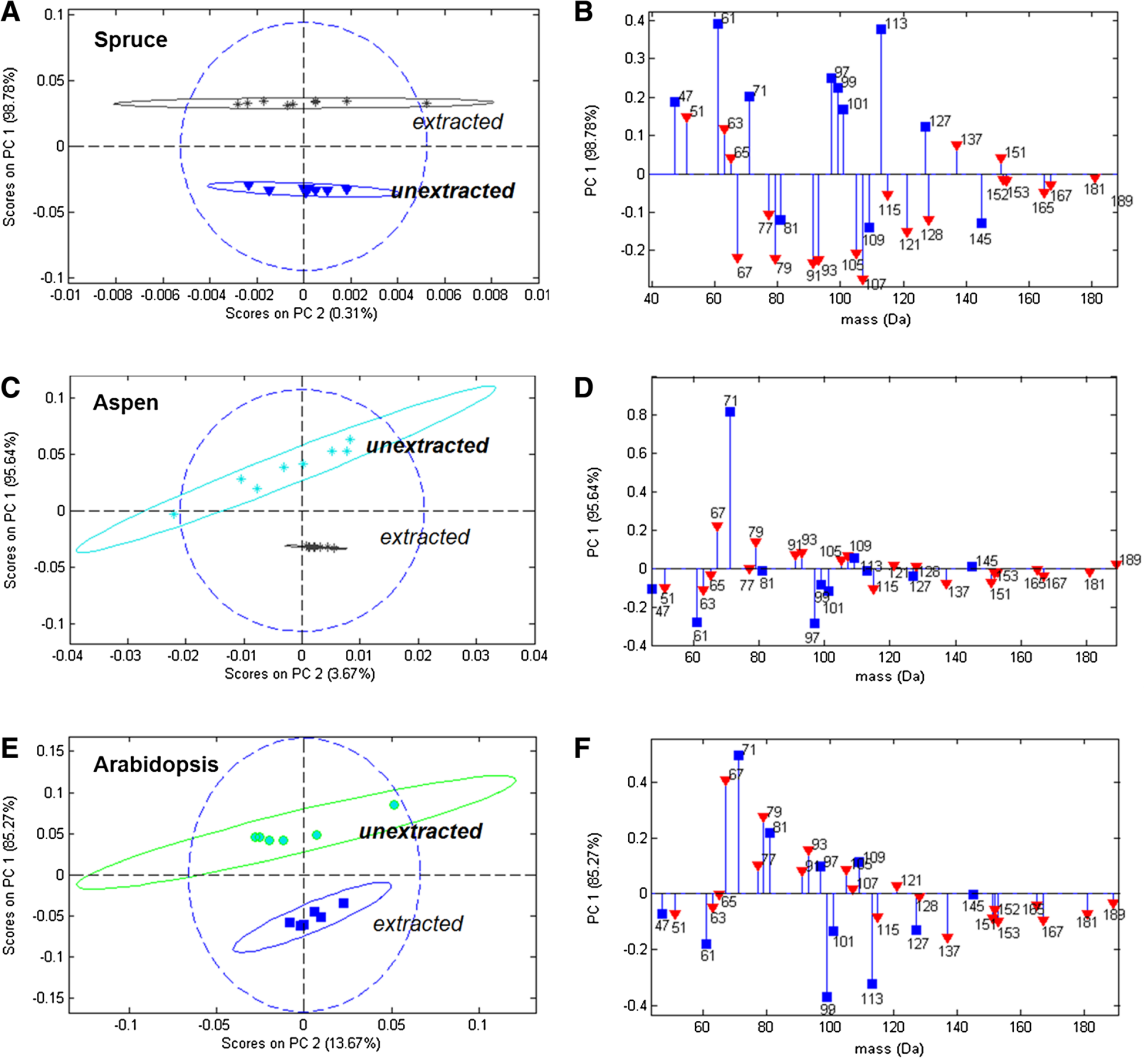
**PCA models showing differences in lignin and polysaccharide-related ToF-SIMS peak intensities due to solvent extraction.** PCA scores **(A**,**C**,**E)** and loadings **(B**,**D**,**F)** for positive ion ToF-SIMS spectra for red spruce sapwood **(A**,**B)**, trembling aspen sapwood **(C**,**D)** and *Arabidopsis thaliana* stem **(E**,**F)** using the list of lignin and polysaccharide-characteristic peaks from Ref [1]. Ellipses in scores plots represent 95% confidence intervals. In the loadings, red triangles denote lignin-related peaks and blue squares denote polysaccharide-related peaks.

Previous ToF-SIMS studies of extractives on pulp [2,7,20] and on wood [12] focused on extractive peaks above 200 Da. Here, the spectra from several unextracted and Soxhlet-extracted lignocellulosic materials (red spruce sapwood, trembling aspen sapwood, and *Arabidopsis thaliana*) were compared to evaluate the influence of extractive compounds on ToF-SIMS spectra below 200 Da, and possible mass overlap of peaks from these compounds with peaks originating from polysaccharides or lignin. While aspen and spruce represent two commercially relevant wood species with different extractive contents, *Arabidopsis thaliana* was included in this analysis as it is an important model plant system used to study cell wall biosynthesis [21]. For the purpose of ToF-SIMS enzyme assays on lignocellulose, PCA comparing extracted and unextracted samples focused on the list of positive ion ToF-SIMS peaks previously used to describe lignin and polysaccharides [1,18] (Figure 2). However, PCA of full spectra from extracted and unextracted samples gave similar results (Additional file 1: Figure S2). PCA highlights the differences in peak patterns between samples using an axis rotation technique [19]. In PCA plots, spectra that score on one side of a principal component (e.g. positive on PC1) are enriched in those peaks which load on the same side of the PC (e.g. positive on PC1) and are depleted in those peaks which load on the other side of the same PC (e.g. negative on PC1).

Although the different plant types exhibited slightly different peak patterns for the unextracted lignocellulose (negative loadings Figure 2B, positive loadings Figure 2D, positive loadings Figure 2F), there were many peaks in common that distinguished unextracted samples from extracted samples. These peaks corresponded with saturated hydrocarbon ions which are also characteristic of fatty acids, and with unsaturated or aromatic hydrocarbon peaks which also characterized lignin in extracted wood [18]. The peaks which most frequently distinguished extracted and unextracted lignocellulose are summarized in Table 2.

**Table 2:** Low-mass ToF-SIMS peaks which generally distinguished unextracted from extracted lignocellulose samples

**Unextracted**			**Extracted**		
**Mass**	**Ion(s)**	**Component(s)**	**Mass**	**Ion(s)**	**Component(s)**
43	C_3_H_7_, C_2_H_3_O	L, PS	29	CHO, C_2_H_5_	PS, L
55	C_4_H_7_, C_3_H_3_O	L, PS	31	CH_3_O	PS
67	C_5_H_7_	L*	45	C_2_H_5_O	PS
71	C_3_H_3_O_2_	PS*	47	CH_3_O_2_	PS*
79	C_6_H_7_	L*	51	C_4_H_3_	L*
81	C_6_H_9_, C_5_H_5_O	L, PS*	59	C_2_H_3_O_2_	PS
91	C_7_H_7_	L*	61	C_2_H_5_O_2_	PS*
93	C_7_H_9_	L*	63	C_5_H_3_	L*
95	C_7_H_11_	L	65	C_5_H_5_	L*
105	C_8_H_9_	L*	69	C_4_H_5_O, C_5_H_9_	PS, L
107	C_8_H_11_, C_7_H_7_O	L*	73	C_3_H_5_O_2_	PS
121	C_7_H_5_O_2_, C_8_H_9_O	L*	85	C_4_H_5_O_2_	PS
128	C_6_H_8_O_3_	L*	97	C_5_H_5_O_2_	PS*
145	C_6_H_9_O_4_	PS*	99	C_5_H_7_O_2_	PS*
			113	C_5_H_5_O_3_	PS*
			127	C_6_H_7_O_3_	PS*
			137	C_8_H_9_O_2_	L*
			151	C_8_H_7_O_3_	L*

Lignin (L) and polysaccharide (PS) designations are from previous publications [1,18]. Those peaks also considered to be free from protein peak interferences are starred* [1].

In this case of deliberate solvent extraction, differences between sample groups illustrated by the scores plots in Figure 2 can be explained by the presence or absence of extractives. It is therefore clear that the peaks in the loadings plots most likely relate to waxes, unsaturated fatty acids, tannins and/or resin acid extractives. However, for non-extracted samples, a key implication of the mass overlap between extractives and lignin is that a change in extractives content could be mistaken for a change in lignin content. The mere presence of extractives decreased the polysaccharide peak fraction [1] from 0.47 ± 0.02 to 0.37 ± 0.02 (Additional file 1: Figure S3A) because of the apparent increase in mostly lignin-related peaks. Extractives also decreased the lignin modification metric [1], which is used to detect lignin degradation, from 0.79 ± 0.02 to 0.56 ± 0.02 (Additional file 1: Figure S3B). This is because the extractives themselves contribute to the intensity of the aromatic peaks in the denominator of this ratio (77 and 91 Da) but not to the lignin-specific peaks in the numerator of this ratio (137, 151, 167 and 181 Da).

Similar to the wood samples, *Arabidopsis thaliana* demonstrated mass overlap of peaks from lignin and extractives that can confound ToF-SIMS analysis of this model plant system. For example, analysis of unextracted Arabidopsis stem from the *irx3* cellulose-deficient mutant initially indicated an unexpected decrease in lignin-related peaks (Additional file 1: Figure S4). However, by comparing the differences in ToF-SIMS spectra between *irx3* and wild-type Arabidopsis, to the differences in ToF-SIMS spectra between extracted and unextracted wild-type stem, it became clear that the apparent change in lignin content in *irx3* stem was instead explained by higher waxy hydrocarbon content in the wild-type plant (Additional file 1: Figure S4).

The current comparison of extracted and unextracted biomass samples confirms that if unextracted samples are to be used, it is critical to have prior knowledge of the expected impacts of applied enzymes or introduced mutations.

### Determination of cellulase dose–response curve

Given that several catalytic activities are required for plant polysaccharide degradation and dissolution, screening of carbohydrate active enzymes (CAZymes) by ToF-SIMS could be achieved by augmenting the activity of a known cellulase cocktail [1]. Moreover, since ToF-SIMS detects fragment ions, cellulose depolymerization alone will not necessarily be detected without subsequent dissolution of the degradation products into the supernatant and their removal by washing. As previously shown [1], detection of glycoside hydrolase activity is then enabled by the enrichment of lignin in the corresponding wood sample.

**Figure 3:**
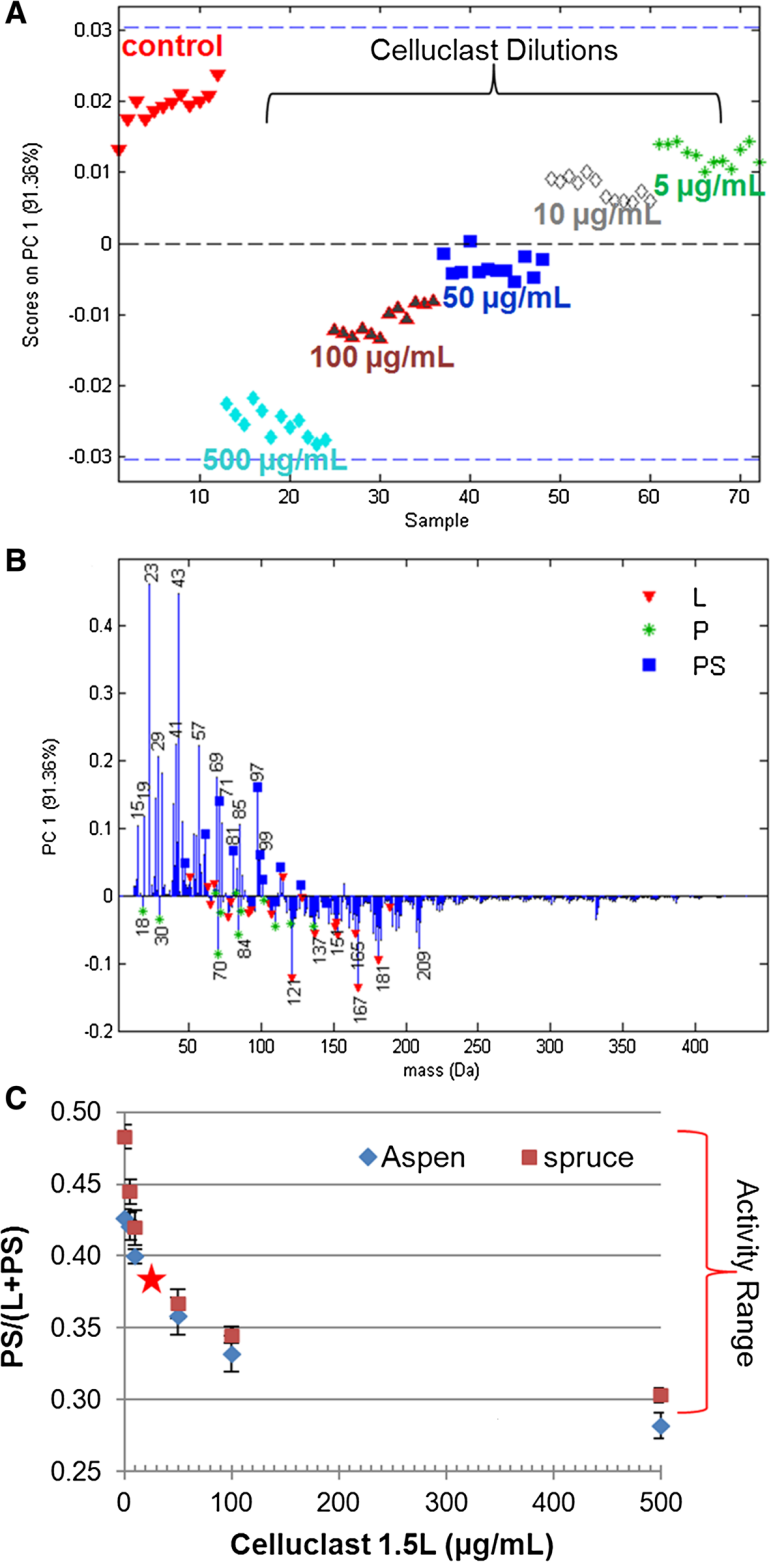
**Establishment of a dose–response curve for extracted trembling aspen treated with Celluclast enzyme dilutions.** PCA scores **(A)** and loadings **(B)** illustrate which peaks in the full spectra changed intensity with cellulase treatment. Peaks in the loadings descriptive of lignin (L), protein (P) and polysaccharides (PS) are denoted by red triangles, green stars and blue squares, respectively. The dose–response curve **(C)** compares the polysaccharide peak fraction [1] against the Celluclast concentration for both extracted red spruce and trembling aspen. Error bars represent one standard deviation (n = 9). The red star estimates the point of 50% surface conversion (50% of the activity range in the PS/(L + PS) polysaccharide peak fraction).

To measure by ToF-SIMS the change in activity of a starting cellulase cocktail upon augmentation with a test enzyme, it is essential that the starting cocktail has not yet fully degraded the sample surface. A range of Celluclast dilutions were therefore incubated with extracted trembling aspen and red spruce (Figure 3 and Additional file 1: Figure S5). Arabidopsis samples were not included in this analysis since this model plant system is not considered a major source of platform sugars.

PCA showed a clear distinction between control aspen powders incubated in buffer alone along with aspen powders incubated in low cellulase concentrations (positive scores Figure 3A, enriched in polysaccharide peaks Figure 3B), against aspen powders incubated in high cellulase concentrations (negative scores, enriched in protein and lignin peaks). Plotting the polysaccharide peak fraction [1] against final Celluclast concentrations revealed a clear dose–response curve (Figure 3C). From this curve, one can estimate that ~20 μg/mL protein should produce 50% of the measureable activity at the surface. Augmentation of the Celluclast cocktail under these experimental conditions should therefore begin with no more than 20 μg/mL of Celluclast.

### Cellulase detection in the presence of extractives

**Figure 4:**
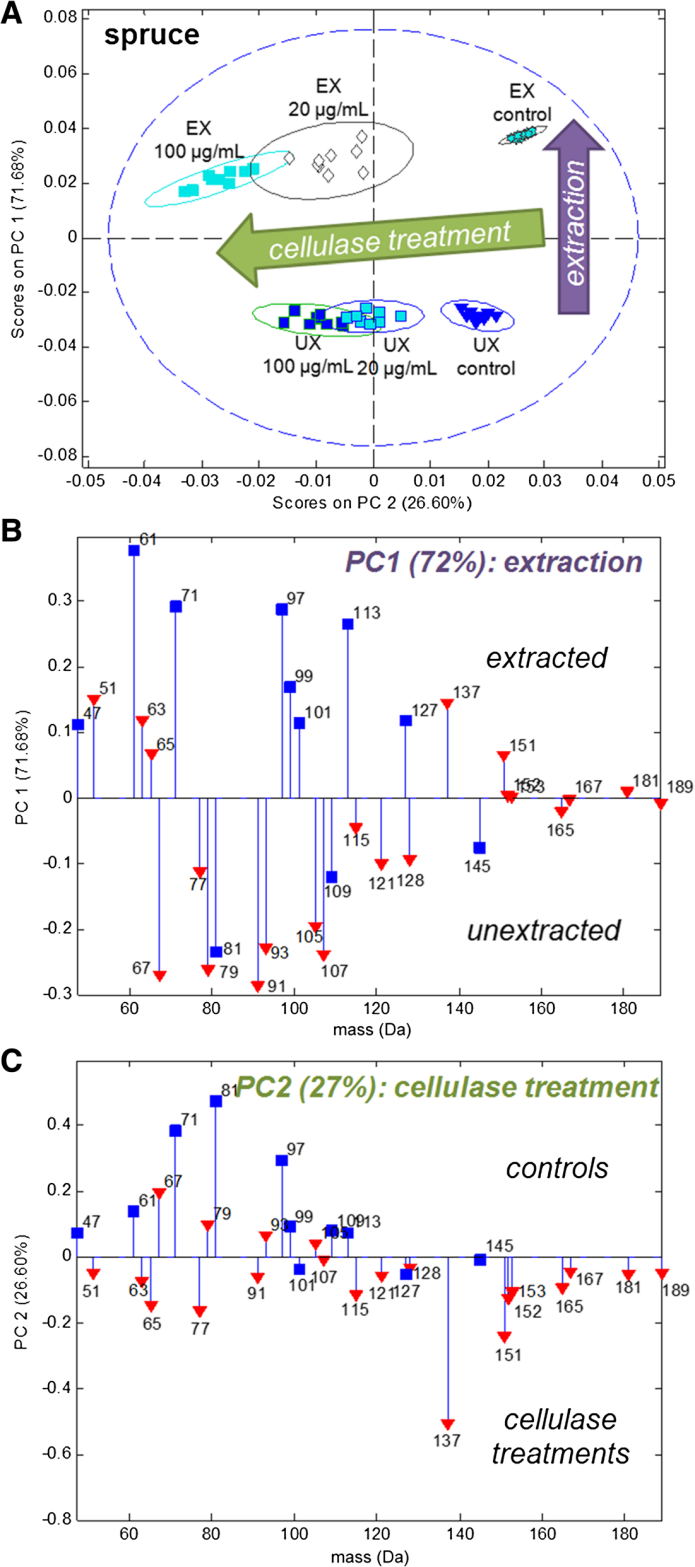
**Distinction of enzyme activity and sample extraction for red spruce in a single PCA model.** PCA scores **(A)** and loadings **(B**,**C)** for a single PCA model describing positive ion ToF-SIMS spectra of extracted and unextracted red spruce incubated with pH 4.9 buffer alone (control) or with added 20 μg/mL or 100 μg/mL Celluclast enzyme (pH 4.9, 50°C, 1 h). Ellipses in scores plots represent 95% confidence intervals. Model was built with the list of lignin and polysaccharide-characteristic peaks from Ref [1]. In loadings, red triangles denote lignin-characteristic peaks and blue squares denote polysaccharide-characteristic peaks.

It is clear from the above discussion that extractives alter the values of peak ratios due to their peak overlap with lignin peaks. However, this does not necessarily prevent the detection of enzyme activity on unextracted wood using multivariate statistical analysis. In fact, when extracted and unextracted red spruce were treated with Celluclast and the resulting positive ion ToF-SIMS spectra were modelled together by PCA, the first two principal components clearly distinguished both the differences in extractives and the changes due to the cellulase enzyme action (Figure 4). This shows that it is indeed possible to detect the enzymatic activity of the Celluclast cocktail even in the presence of extractives.

PC1 of this model described 71.68% of the variance in the data set and separated extracted samples (positive scores) from unextracted samples (negative scores) according to the same peak pattern for spruce extractives that was observed in Figure 2. PC2 described 26.60% of the variance and separated the untreated control samples (positive scores) from the cellulase-treated samples, which progressed from neutral to negative scores with increasing enzyme dose. Notably, 20 μg/mL treatments were approximately half-way between the controls and the 100 μg/mL treatment point for these incubation conditions, as predicted from the dose–response curve in Figure 3C. The cellulase treatments on the extracted and unextracted spruce sample subsets were also modelled separately (Additional file 1: Figure S6) and these models independently produced nearly identical loadings patterns to Figure 4C as a result of the cellulase treatments.

Several important observations may be made by comparing cellulase action on extracted and unextracted spruce samples. First, the spread between the PC2 scores of the controls and the 100 μg/mL treatments was smaller for the unextracted spruce than for the extracted spruce (Figure 4A). There are several possibilities for why enzyme action on unextracted spruce appears to be decreased. First, it is possible that the extractives coat the lignocellulose fibers, decreasing the observed lignocellulose ion intensity, thereby making these ions a smaller portion of the total spectrum with proportionally smaller changes. Second, it is possible that the extractives are inhibiting the enzyme action. Both mechanisms, and other factors, may be at play and merit further investigation.

Furthermore, while earlier ToF-SIMS analyses of cellulase activity on spruce samples distinguished control and enzyme treated samples by unambiguous enrichment of polysaccharide and lignin peaks, respectively [1], in the present study, the PC2 loadings plot described the control samples by both polysaccharide and lignin-descriptive peaks (Figure 4C). Notably, the lignin peaks describing the control samples were at 67, 79, 93 and 105 Da, which also represent unsaturated (but not aromatic) hydrocarbon peaks (Table 2). The cause of this loading pattern is unclear, but might be due to the inclusion of unextracted samples in this PCA model, or dissolution of extractive compounds upon enzyme treatment.

The differences in peak patterns in PC1 and PC2 loading plots suggest that peaks originating from lignin or extractive compounds might be distinguished by the relative intensities of confounding peaks (Figure 4B,C). In this case, it is clear that considering the data using multivariate analysis such as PCA is a much more powerful approach than computing raw peak intensity ratios. For instance, PCA (or other MVA techniques) can be used to separate the effects of extractives and enzymes, allowing scores on the enzyme-related component to be used alone for relative quantification.

To use this approach, chemical standards or unextracted and extracted biomass samples must be prepared. However, by examining the loadings in Figure 4C, the following guidelines could be applied to predict differences in spruce samples that result from differences in lignin content: 1) the 137 and 151 Da peaks are on the same side of the loadings plot as the 65, 77 and 91 Da aromatic peaks; and 2) on the side of the loadings plot that has the 137 and 151 Da peaks, the 77 and 91 Da peaks (aromatic) load more strongly than the 79 and 93 Da peaks (unsaturated, non-aromatic), respectively. If the opposite statements are true, then differences between samples are more likely due to differences in extractives content (e.g. in PC1 (Figure 4B), the 137 and 151 Da peaks are on opposite sides of the loadings from the 65, 77 and 91 Da aromatic peaks and the 79 Da peak loads more strongly than the 77 Da peak.)

Critically, when discussing issues of peak intensities and mass overlap, it is always important to recall a few fundamental aspects of the ToF-SIMS measurement. First, it is important to note that the use of different primary ion sources will change the intensity patterns of secondary ion peaks, with gentler sources (e.g. large argon clusters [22]) generally producing fewer fragment ions and higher molecular ion signals. It is also important to note that all of the data presented here were processed using nominal mass binning due to sample roughness. The peak patterns observed will depend on the abundance of multiple peaks contained within a nominal mass (e.g. for mass 81 Da) and therefore on sample chemistry. Certainly, richer detail on those peaks related to extractives and lignocellulose would be obtained if better mass resolution is used. An intriguing prospect for achieving better mass resolution on topographically challenging samples is the data post-processing recently published by Pachuta and Vlasak [23].

### Extractives and cellulase augmentation

**Figure 5:**
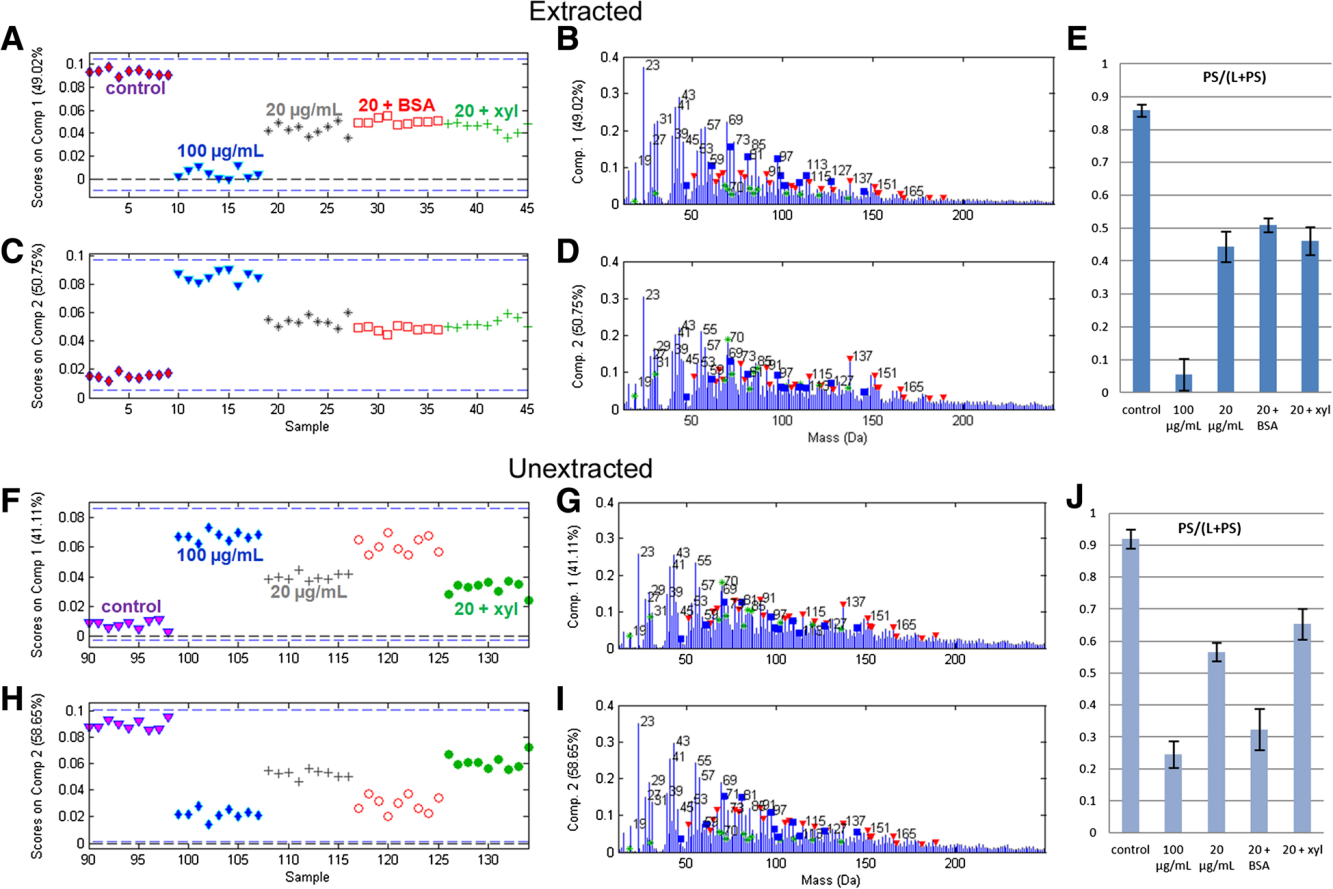
**Effects of augmenting the 20 μg/mL cellulase treatment with BSA or with xylanase (xyl).** Panels **A-E** show results of a two-curve MCR model of extracted red spruce and panels **F-J** show results of a two-curve MCR model of unextracted red spruce. Scores **(A,C,F and H)** show the position of each sample on a continuum between the control and the 100 μg/mL cellulase treatment. In loadings plots **(B,D,G and I)** lignin-related peaks are denoted by red triangles, protein-related peaks by green stars, and polysaccharide-related peaks by blue squares. In panels **E** and **J**, the ratios of the scores are calculated to provide a polysaccharide peak fraction (note that this is a different method than taking a ratio of raw peak intensities). Error bars represent one standard deviation (n = 9).

To explore the idea of screening proteins by cellulase cocktail augmentation, samples were made by spiking the 20 μg/mL Celluclast cocktail with either BSA as a control that has no catalytic activity on wood, or with a commercial xylanase enzyme. The results of the cellulase augmentation experiment on spruce were well-visualized with simple two-component MCR models (Figure 5). In these models, the scores on comp. 1 and comp. 2 had inverse patterns from each other, as expected. For both extracted and unextracted spruce, the loadings showed increased polysaccharide peak intensities for the component on which the control samples scored highly, contrasted against increased protein and lignin peak intensities for the other component on which the 100 μg/mL cellulase treatments scored highly. Slight differences in loading peak intensities were observed between the extracted and unextracted spruce but the overall form of the component spectra were similar. The cellulase augmentation results are also visualized using ratios of the scores in Figure 5E,J, which provide a clearer description of the data than do the ratios of raw peak intensities provided in Additional file 1: Figure S3A.

Examining the scores plots, augmentation results were unremarkable for extracted spruce (Figure 5A,C) since extracted spruce treated with 20 μg/mL Celluclast plus BSA or xylanase had similar scores to spruce treated with 20 μg/mL Celluclast alone. On the other hand, scores for unextracted spruce showed that spiking the 20 μg/mL cellulase with the inert BSA protein increased the cellulolytic activity, with the scores of these samples shifted towards the stronger 100 μg/mL cellulase treatment (Figure 5F,H). This result highlights the differences in enzyme activity in the presence or absence of extractives and is consistent with the possibility that the presence of BSA could minimize cellulase inhibition or non-productive associations to unextracted wood samples ([24,25]). In contrast to BSA, which seemed to enhance cellulolytic activity on unextracted spruce, the addition of xylanase seemed to shift the spectra scores towards the controls on unextracted spruce (Figure 5F,H). Although the amount of xylan in spruce is low, this result might indicate that xylanase action in unextracted samples led to cleavage of more bonds to lignin. In this case, the release of both lignin and polysaccharides would reduce the relative change in polysaccharide and lignin content measured by ToF-SIMS.

These results confirm that cellulase cocktail augmentation may be studied by ToF-SIMS, as well as more fundamental comparisons of enzyme action on extracted and unextracted lignocellulosic substrates.

## Conclusions

Several advances were made towards increasing the throughput and practicality of ToF-SIMS enzyme assays for lignocellulosic materials. It was found that 96-well filter plates provided contamination-free, rapid processing of samples for fiber-based enzyme assays using ToF-SIMS. Such plates can be used with either manual or robotic processing. The spectral overlap between extractives and structural polymers significantly altered peak ratios established for extracted wood, mainly due to the overlap between extractives and lignin-characteristic peaks. Due to this, peak ratios based on selected lists could lead to erroneous conclusions. However, multivariate statistical methods revealed enzyme activity in the presence of extractives. It is suggested that multivariate methods be used whenever possible to detect contaminants and to unbiasedly reveal informative patterns within the data. Lastly, augmentation of cellulase activity by other proteins can be detected by ToF-SIMS, although not surprising, the impact of the augmentation will likely be influenced by the presence of extractives.

## Methods

### Plant species

Softwood from red spruce (*Ricea rubens*) and hardwood from trembling aspen (*Populus tremuloides*) were obtained and milled as described in Ref [1]. Milled powders passed through a US mesh size 100 sieve (0.150 mm diameter). Sapwood was used for all assays. Mature wild type *Arabidopsis thaliana* stem from 30 mature plants was harvested, pooled and homogenized with a Mini-Beadbeater-16 (Biospec Products, Bartlesville, USA).

### Soxhlet extraction

After milling, portions of aspen and spruce were separately extracted to remove low molecular weight, non-structural wood extractives such as resin acids, fatty acids, terpenes, tannins and hydrocarbons using ethanol, toluene, and water extraction by ASTM Standard D1105-96 (2007). *Arabidopsis thaliana* stem samples were extracted in 95% (v/v) ethanol for 4–5 h, followed by ethanol-toluene (2:1) for 6–8 h, and boiling water for 3 h. Washed samples were air dried for at least four days before being used.

### Dispensing wood samples to 96-well filter plates and testing for sample contamination

The 96-well filter plates were Millipore MultiScreen Barex/TiO_2_ plates (catalog number MSBVN1B50). The plates have 1.2 μm Durapore PVDF membrane filters individually sealed at the well bottoms, with a removable under-drain allowing for suction of liquid in the wells out through the bottom of the plate using a vacuum manifold.

The mass of wood powder required to cover a given area of Scotch® double-sided adhesive tape was measured and used to calculate the amount of wood powder required in each well in order to completely cover the tape during transfer of wood powder from the 96-well plate for ToF-SIMS analysis. Although it was calculated that ~2 mg of <100 mesh wood was required to cover the bottom of the plate, 4–5 mg of wood was used to ensure sufficient coverage to prevent the PVDF membrane from sticking to the tape and to allow for sample mass loss due to enzymatic degradation.

Filter plates were initially checked for contamination of the wood samples as follows. All but three wells were masked with tape, the three open wells were pre-rinsed by soaking for 1 min with pH 4.9 Britton-Robinson universal buffer [1] and the buffer was removed with vacuum suction. 5–10 mg dry extracted red spruce was dispensed into each well, 250 μL pH 4.9 buffer was added to each well and the plate was incubated at 37°C for 2 hours. The supernatant was then filtered through and discarded, and the wood was rinsed with ~30 mL of MilliQ water per well under continuous vacuum suction. In later experiments, higher incubation temperatures were used (e.g. 1 h at 55°C) and rinsing used discrete aliquots (e.g. 10× 250 μL) of MilliQ water in order to re-suspend the wood and give more time for adsorbed buffer salts to dissolve in the rinse water. After washing, the wood was air dried and transferred to tape as shown in Additional file 1: Figure S1 for ToF-SIMS analysis.

### Cellulase dose–response curve

A commercial cellulase enzyme cocktail was chosen to target cellulose hydrolysis. The cellulase stock solution (50 mg protein/mL) consisted of 10 FPU (filter paper unit) Novozym Celluclast 1.5L (endo-1,4-β-glucanases and cellobiohydrolases) (Sigma-Alrich, St. Louis, USA). A dose–response curve was generated by incubating extracted red spruce and trembling aspen with different dilutions of the Celluclast enzyme cocktail. A 20 μg/mL wood particle slurry was made with pH 4.9 universal buffer and stirred vigorously prior to pipetting 200 μL of slurry into each well of a 96-well filter plate. From the Celluclast stock, 2000×, 1000×, 200×, 100× and 20× dilutions were prepared in pH 4.9 universal buffer and 50 μL of each dilution was added to each wood in duplicate. This produced treatments that had 1.6% solids loading (4 mg wood per 250 μL) and net 5, 10, 50, 100 and 500 μg/mL cellulase. Buffer controls with no enzyme were also made. All samples were incubated for 1 h at 55°C with intermittent shaking. The supernatant was then filtered through the plate, and the solids were washed with 10× 250 μL portions of room-temperature MilliQ water, allowing each portion to soak with the wood for approximately 1 minute. Samples were then air-dried at room temperature overnight and transferred to tape for ToF-SIMS analysis.

### Cellulase augmentation and extractive assays

Wood slurries of unextracted and extracted red spruce were prepared at 25 mg/mL in pH 4.9 universal buffer. 200 μL of wood slurry was dispensed into each well of a 96-well filter plate to provide 5 mg wood per well and 2.0% solids loading upon addition of enzymes to 250 μL total volume. Control samples were prepared using wood and buffer alone. Cellulase treatments consisted of 100 μg/mL Celluclast, 20 μg/mL Celluclast, and 20 μg/mL Celluclast augmented with either 20 μg/mL xylanase (Novozym 51024) or 20 μg/mL bovine serum albumin (BSA). Duplicate wells were prepared for each treatment. The plate was incubated for 1 h at 50°C in a shaker-incubator at 120 rpm. After incubation, 100 μL of each duplicate well was pooled together to reduce variability. Samples were washed with 10× 250 μL MilliQ water with 1 minute rest time for each rinse. The samples were then air-dried overnight and transferred to tape for ToF-SIMS analysis.

### Time-of-flight secondary ion mass spectrometry

ToF-SIMS measurements were made with a ToF-SIMS IV instrument (Ion-Tof Gmbh, Münster, Germany) equipped with a bismuth liquid metal ion source and reflectron-type analyzer with multichannel detector. Spectra were acquired using 50 keV Bi_3_^2+^ primary ions (~0.3 pA pulsed current) incident at 45°, operated on a 100 μs cycle time with high-current bunched conditions. Spectra were acquired for between 45 and 90 s using a 128 × 128 pixel random raster pattern covering a 500 × 500 μm^2^ area. Ion doses were kept below 1 × 10^12^ ions/cm^2^ to limit sample damage. To ensure adequate sampling for each treatment, at least 6 positive ion spectra were acquired for each treatment. The pressure during analysis was maintained between 1×10^-8^ and 1×10^-7^ mbar. Low energy electron flooding (20 eV) was used to reduce sample charging. Positive ion spectra were calibrated to CH_3_^+^, C_2_H_3_^+^ and C_3_H_5_^+^ ions using SurfaceLab v.6.1 software. Mass resolution (M/ΔM) varied depending on sample roughness and all ToF-SIMS data were binned to 1 Da before calculating peak ratios or applying statistical analysis.

### Principal component analysis and multivariate curve resolution

PCA and MCR were performed using Matlab software v.8.0.0.783 R2012b (The Mathworks, Inc.) with PLS Toolbox v.7.0.3 (Eigenvector Research Inc.). Individual ToF-SIMS spectra from replicate locations on the wood powder were preprocessed as follows. Poisson scaling (square root mean scaling) was applied to adjust for the inherent skew in SIMS data towards lower peak intensities at higher mass (and increased noise at these higher masses) [26]. Then, the peak intensities were normalized to the total ion intensity of the peak list in order to eliminate the influence of overall intensity changes that might arise from topography or variations in instrumental setup, such as primary ion dose or acquisition time. Lastly, for PCA analysis, data at each mass was also mean centered so that all PCs described variations from the data set mean. Due to the non-negativity requirement of MCR, data was not mean centered for MCR. The number of component curves for MCR was established by preliminary PCA models.

## Abbreviations

CAZymes: Carbohydrate active enzymes; MCR: Multivariate curve resolution; MVA: Multivariate analysis; PC: Principal component; PCA: Principal component analysis; PDMS: poly(dimethyl siloxane); ToF-SIMS: Time-of-flight secondary ion mass spectrometry.

## Competing interests

The authors declare that they have no competing interests.

## Authors’ contributions

RG, AT and EM designed the study. RG performed all experiments related to spruce and aspen. AT performed the Arabidopsis experiments. RG drafted the manuscript. All authors reviewed and approved the final manuscript.

## Supplementary Material

Additional file 1: Table S1 Consequences of tape show-through on peak ratios. **Figure S1.** Transfer of wood powder from a 96-well filter plate to tape for ToF-SIMS analysis. **Figure S2.** PCA models showing differences due to solvent extraction using all ToF-SIMS peaks between 12 and 450 Da. **Figure S3.** Effect of extraction on the polysaccharide peak fraction and lignin modification metric. **Figure S4.** PCA comparing unextracted wild-type and unextracted mutant *Arabidopsis*, and comparing extracted and unextracted wild-type *Arabidopsis*. **Figure S5.** PCA scores and loadings for the treatment of extracted red spruce with different dilutions of Celluclast enzyme. **Figure S6.** Cellulase activity on extracted and unextracted red spruce described in two separate PCA models. Click here for file
