# Supplementary material for: Towards practical time-of-flight secondary ion mass spectrometry lignocellulolytic enzyme assays
Source: Biotechnol Biofuels. 2013 Sep 14;6:132. doi: 10.1186/1754-6834-6-132 (PMC3847459; doi:10.1186/1754-6834-6-132)
Supplement: Additional file 1: Table S1 — Consequences of tape show-through on peak ratios. Figure S1. Transfer of wood powder from a 96-well filter plate to tape for ToF-SIMS analysis. Figure S2. PCA models showing differences due to solvent extraction using all ToF-SIMS peaks between 12 and 450 Da. Figure S3. Effect of extraction on the polysaccharide peak fraction and lignin modification metric. Figure S4. PCA comparing unextracted wild-type and unextracted mutant Arabidopsis, and comparing extracted and unextracted wild-type Arabidopsis. Figure S5. PCA scores and loadings for the treatment of extracted red spruce with different dilutions of Celluclast enzyme. Figure S6. Cellulase activity on extracted and unextracted red spruce described in two separate PCA models. [file 1754-6834-6-132-S1.pdf]

Supporting information

Towards practical ToF-SIMS lignocellulolytic enzyme assays

Robyn E Goacher, Alex Yi-Lin Tsai, Emma R Master

Table S1 Consequences of Tape Show-Through on Peak Ratios. MCR scores (from Figure 1 of the main text) for each of the 15 replicate spectra of Soxhlet-extracted red spruce spread on adhesive tape, along with the calculated peak ratios from Ref. [1] for the same spectra. The scores and peak ratios are color-coded so that the smallest values are green and the largest values are red.

	**Buffer Salts**	**PDMS**	**Tape - 1**	**Extracted Wood**	**Tape - 2**		
	Scores on Comp 1 (31.74%)	Scores on Comp 2 (1.93%)	Scores on Comp 3 (13.06%)	Scores on Comp 4 (28.27%)	Scores on Comp 5 (24.63%)	**PS/(L+PS)**	**G+S/Ar**
ExS-p1_0	0.00051	0.006447	0.002106	0.07793	0.017353	0.5007	0.9209
ExS-p2_0	0.001016	0.005571	0.005834	0.070154	0.028186	0.5138	0.8507
ExS-p3_0	0.000119	0.006194	0.000944	0.081479	0.018096	0.5183	0.9303
ExS-p4_0	0.000568	0.006547	0.00269	0.076658	0.022833	0.5205	0.9012
ExS-p5_0	0	0.00603	0.002913	0.07952	0.023169	0.5327	0.8779
ExS-p6_0	0	0.007206	0	0.080594	0.015056	0.5029	0.9904
ExS-p7_0	0.000166	0.006329	0.010912	0.057952	0.043032	0.5432	0.7714
ExS-p8_0	0.001138	0.006725	0.00707	0.069931	0.029214	0.5396	0.8381
ExS-p9_0	0.002634	0.006342	0.011152	0.059406	0.038789	0.5395	0.7989
ExS-p10_0	0.001284	0.007724	0.013519	0.053208	0.049135	0.5668	0.7277
ExS-p11_0	0.002658	0.006886	0.01767	0.04649	0.05436	0.5601	0.6817
ExS-p12_0	0.007311	0.072213	0.007235	0.029663	0.002209	0.5424	0.7823
ExS-p13_0	0.003433	0.036889	0.005801	0.052968	0.015329	0.5454	0.8087
ExS-p14_0	0.002986	0.02534	0.007144	0.056112	0.021021	0.5253	0.8189
ExS-p15_0	0.000891	0.007476	0.003634	0.071215	0.024946	0.5138	0.8774
					Avg	0.5310	0.8384
					Stdev	0.0196	0.0814
					% RSD	3.7%	9.7%

As illustrated in Table S-1, the scores on the tape components in Figure 1 (Comp. 3 and Comp 5) are correlated with an increase in the polysaccharide peak fraction denoted PS/(L+PS) and with a decrease in the lignin modification metric denoted (G+S)/Ar. Although variability is observed between te 15 replicate spectra on all components, it is notable that the other major contaminant of concern - PDMS - does not appear to dominate the changes in these peak ratios. This is encouraging since these peak ratios were chosen to deliberately exclude the effects of PDMS contamination.

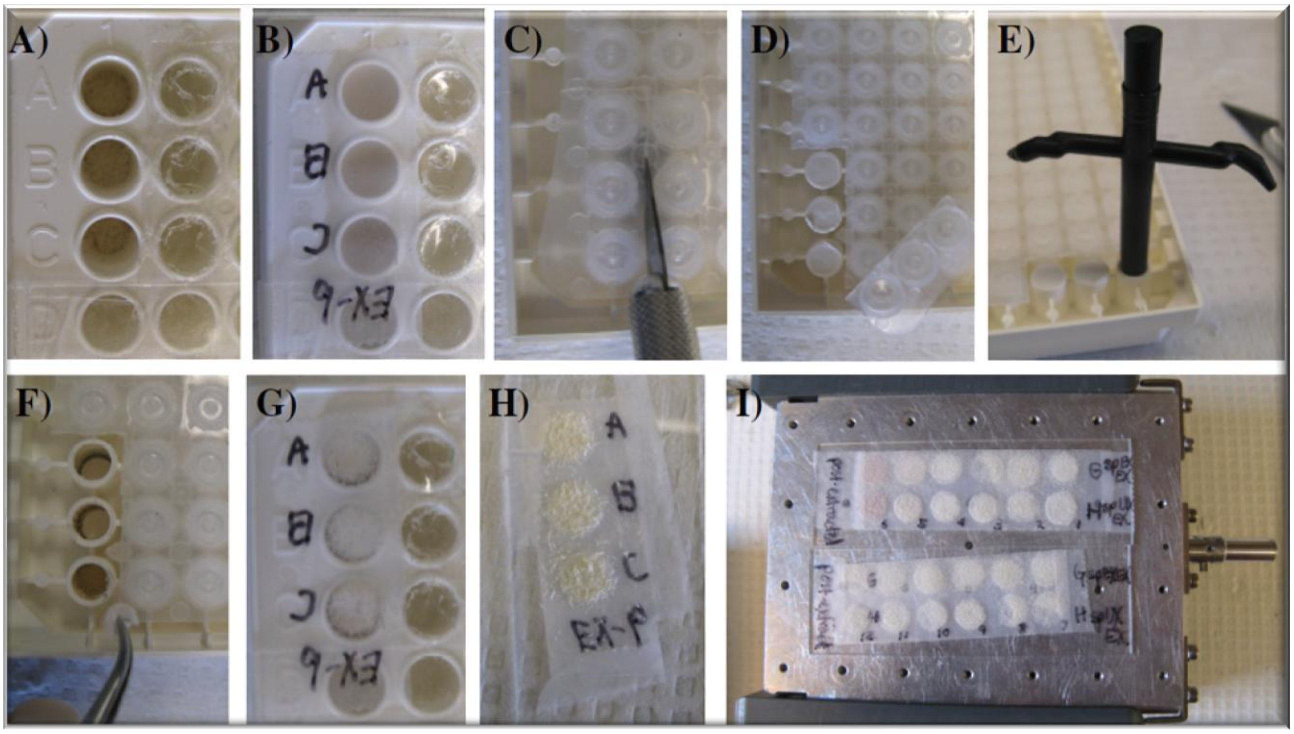

Figure S1 Transfer of wood powder from a 96-well filter plate to tape for ToF-SIMS analysis.

A) Air-dried wood in wells A1-C1 viewed from the top of the filter plate.

B) Scotch tape is placed over the wells containing wood and the tape is lettered backwards.

C) Filter plate is flipped upside down and the under-drain around wells of interest is cut (only necessary if plate is partially used and remainder of plate is to be re-used).

D) The cut under-drain is removed to reveal the PVDF membranes on the well bottoms.

E) Membranes are punched through from back to front using an acetone-cleaned plastic puncher and wood is pressed firmly onto the Scotch tape.

F) After punching, membranes are removed using clean tweezers.

G) The plate is turned upright again and wood fibers are visible, adhered to the Scotch tape on the plate face. Excess fibers are tapped loose.

H) The Scotch tape is removed from plate face and adhered to a glass slide with wood side up.

I) Glass slides are mounted for introduction into the ToF-SIMS. Optimized geometries can allow 48 samples to be introduced at once. Loadlock pumping time is typically ~1 hour.

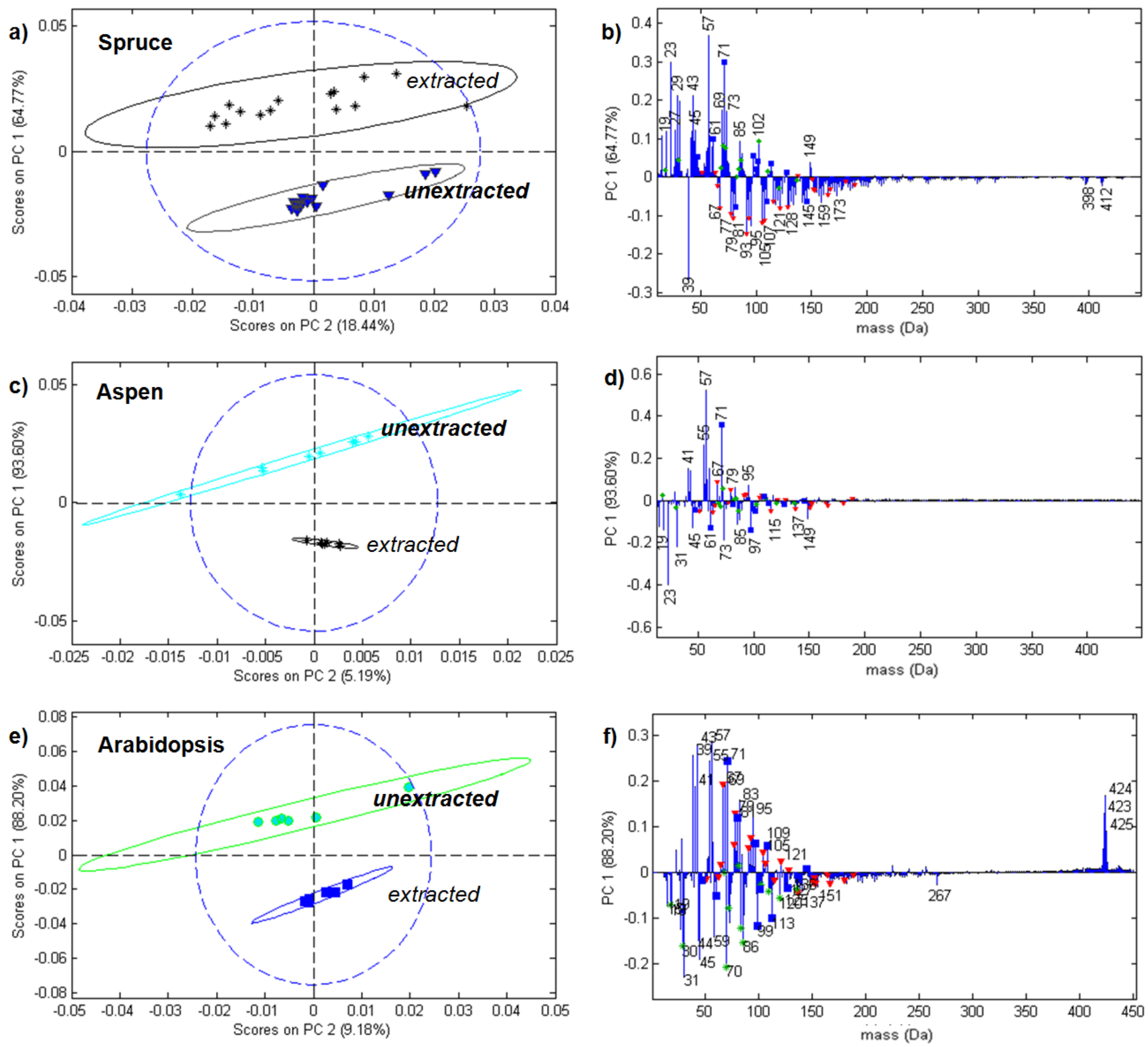

Figure S2 PCA models showing differences due to solvent extraction using all ToF-SIMS peaks between 12 and 450 Da. PCA scores (A, C, E) and loadings (B, D, F) for positive ion ToF-SIMS spectra for red spruce sapwood (A, B), trembling aspen sapwood (C, D) and *Arabidopsis thaliana* stem (E, F) using the full spectra. Ellipses in scores plots represent 95% confidence intervals. In the loadings, red triangles denote lignin-related peaks, green stars denote protein-related peaks, and blue squares denote polysaccharide-related peaks.

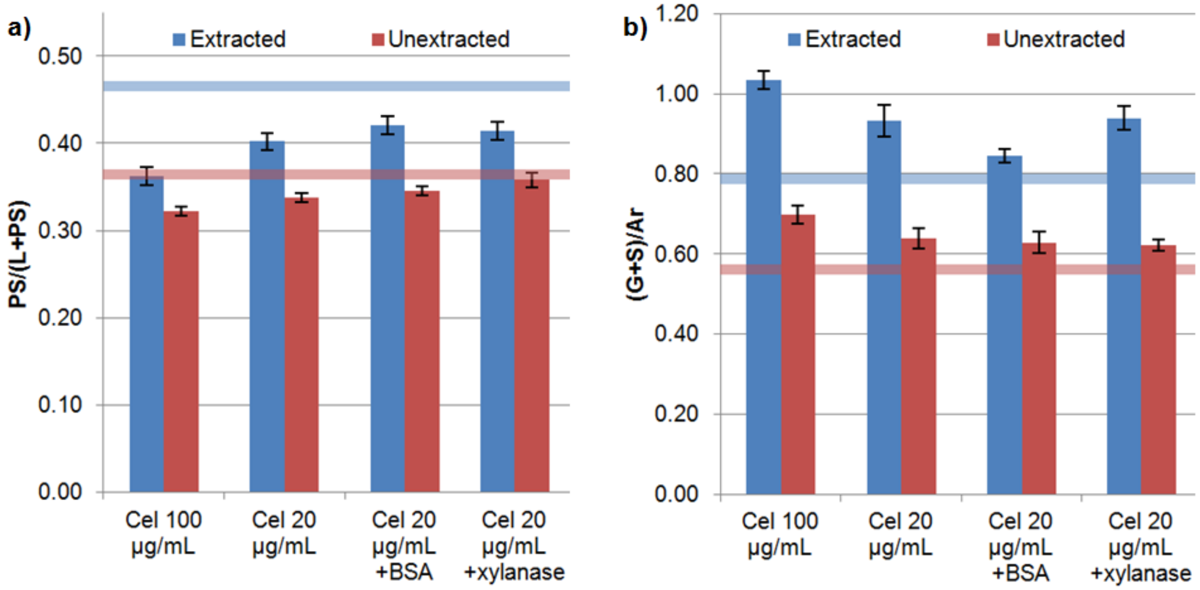

Figure S3 Effect of extraction on the polysaccharide peak fraction and lignin modification metric. The polysaccharide peak fraction (A) and lignin modification metric (B) calculated for unextracted (red) and extracted (blue) red spruce treated with different quantities of cellulase, bovine serum albumin (BSA) and xylanase. Shaded horizontal bars represent the values for the control samples soaked in buffer without any protein. The height of the shaded bars and the error bars represent one standard deviation (n=9). Data are from the same samples represent in Figure 5 of the main text.

For the polysaccharide peak fraction (Fig S-3A), bars reaching below their respective shaded buffer control lines indicate treatments where polysaccharides were degraded and dissolved away by the cellulase enzymes. Comparing the shaded lines representing the controls, the unextracted spruce (red shaded line) is interpreted to have significantly less polysaccharides than the extracted spruce (blue shaded line), but this is an artifact of the overlap between extractives and lignin. Extractives decrease the apparent PS amounts since they mainly overlap with lignin, appearing to increase lignin. Extractives also appear to change how many enzyme treatments appear active since the 20 µg/mL Celluclast enzyme cocktail with xylanase appears active on extracted spruce but not on unextracted spruce.

For the lignin modification metric (Fig S-3B), bars above the buffer control line seem to indicate an enrichment in lignin. It is notable that all the values for unextracted wood are lower than for the extracted wood since the extractives themselves contribute to the intensity of the aromatic peaks in the denominator of this ratio (77 and 91 Da) but not to the lignin-specific peaks in the numerator of this ratio (137, 151, 167 and 181 Da). A difference in the change from the control is also evident for the 20 µg/mL Celluclast with xylanase using this metric.

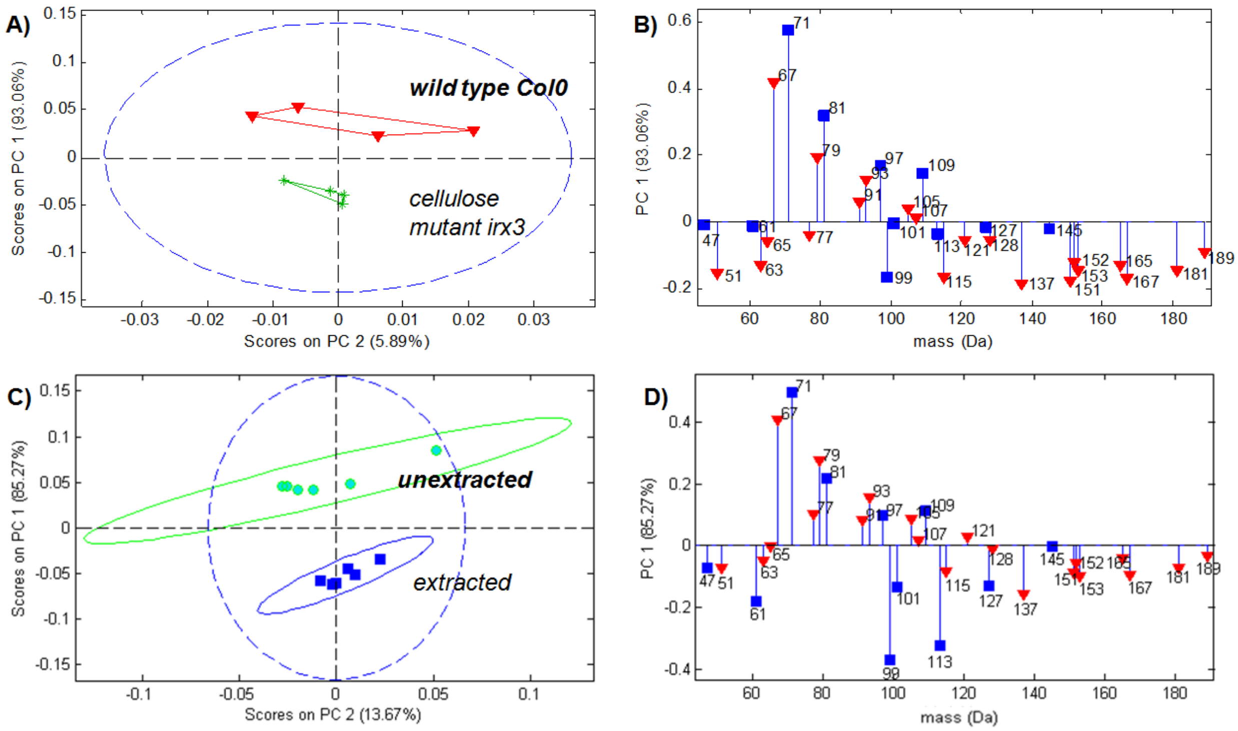

Figure S4 PCA comparing unextracted wild-type and unextracted mutant *Arabidopsis*, and comparing extracted and unextracted wild-type *Arabidopsis*. Panels A and B show the scores and loadings for the distinction of unextracted wild-type *Arabidopsis* (Col0) from the unextracted cellulose mutant (irx3). Panels C and D show the scores and loadings for the distinction of extracted and unextracted wild-type *Arabidopsis*. Note the similar positive loadings pattern for both separations, indicating that extractives play a significant role in the distinction between the mutant and wild-type plants.

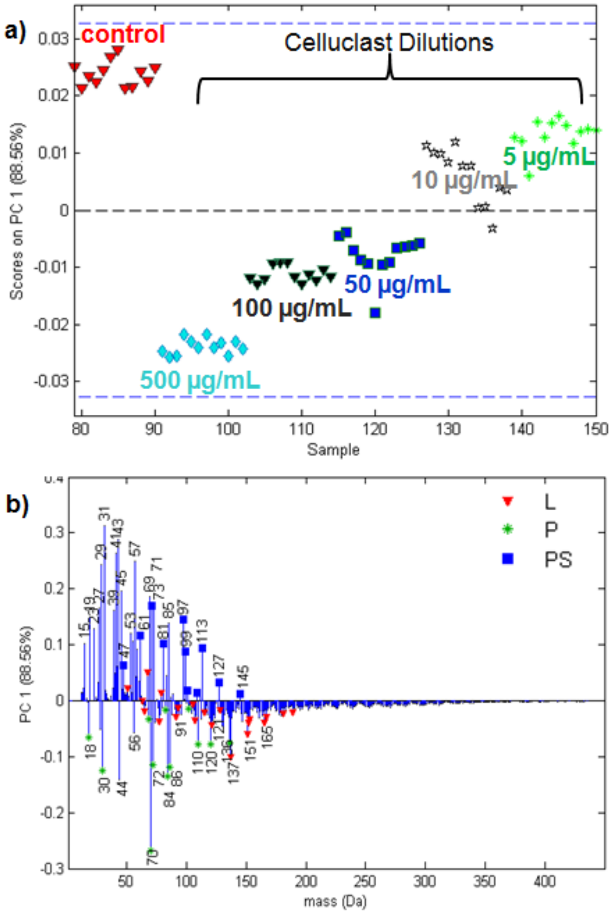

Figure S5 PCA scores (a) and loadings (b) for the treatment of extracted red spruce with different dilutions of Celluclast enzyme (pH 4.9, 55°C, 1 h). The most important difference versus the aspen data shown in Figure 3 of the main text is the absence of S-lignin peaks at 167 and 181 Da in this spruce data.

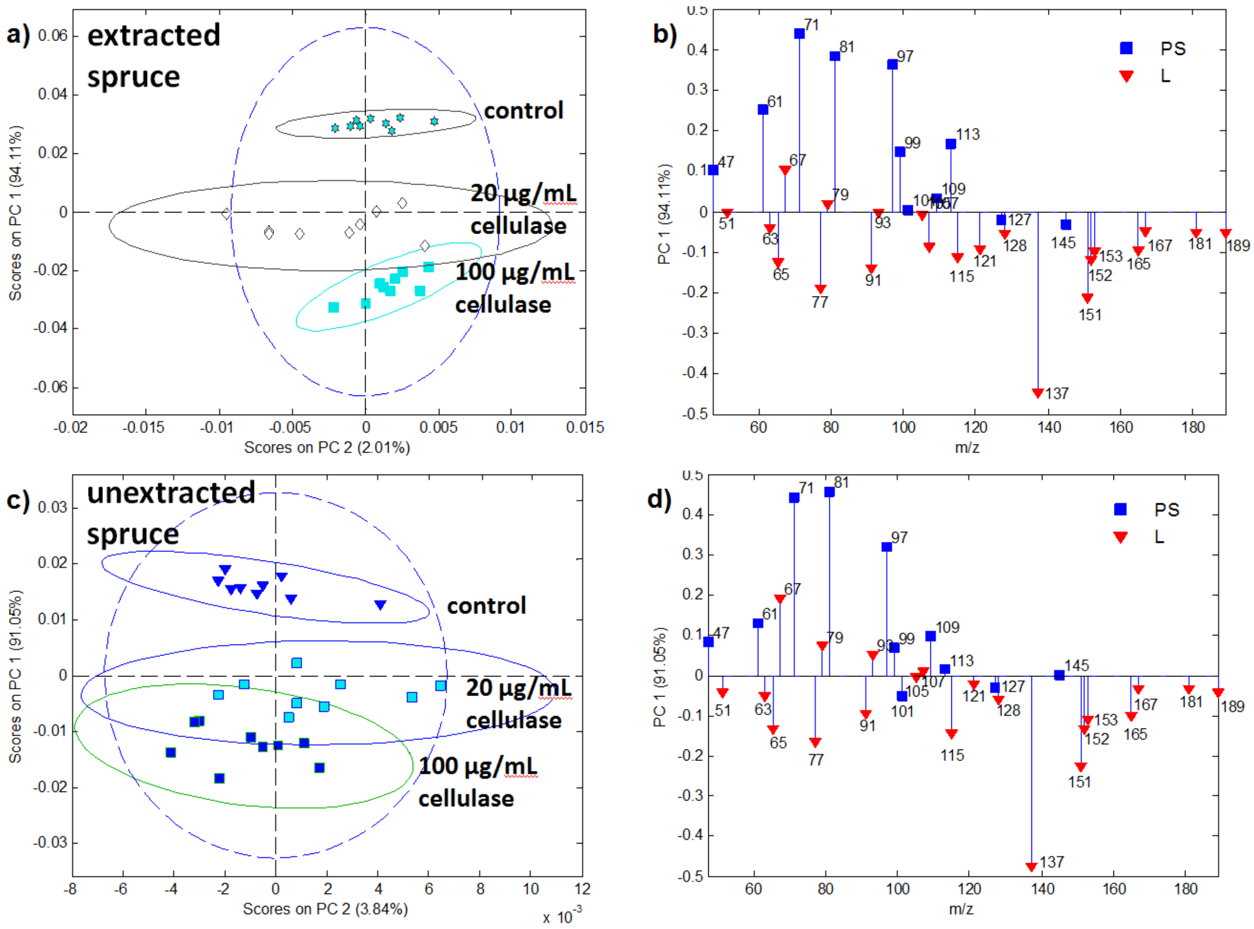

Figure S6 Cellulase activity on extracted and unextracted red spruce described in two separate PCA models. PCA scores (A, C) and loadings (B, D) for two separate models describing extracted (A-B) and unextracted (C-D) red spruce incubated with pH 4.9 buffer alone (control) or with added 20 µg/mL or 100 µg/mL Celluclast enzyme (pH 4.9, 50°C, 1 h). Ellipses in scores plots represent 95% confidence intervals. Model was built with the list of lignin and polysaccharide-characteristic peaks from Ref [1]. In loadings, red triangles denote lignin-characteristic peaks and blue squares denote polysaccharide-characteristic peaks.
